# Integration of Spatial Probability and Size in Slope-Unit-Based Landslide Susceptibility Assessment: A Case Study

**DOI:** 10.3390/ijerph17218055

**Published:** 2020-11-01

**Authors:** Langping Li, Hengxing Lan

**Affiliations:** 1 State Key Laboratory of Resources and Environmental Information System, Institute of Geographic Sciences and Natural Resources Research, Chinese Academy of Sciences, Beijing 100101, China; lanhx@lreis.ac.cn; 2 School of Geological Engineering and Geomatics, Chang’an University, Xi’an 710064, China

**Keywords:** landslide susceptibility assessment, slope-unit, spatial probability, size, integration

## Abstract

Landslide spatial probability and size are two essential components of landslide susceptibility. However, in existing slope-unit-based landslide susceptibility assessment methods, landslide size has not been explicitly considered. This paper developed a novel slope-unit based approach for landslide susceptibility assessment that explicitly incorporates landslide size. This novel approach integrates the predicted occurrence probability (spatial probability) of landslides and predicted size (area) of potential landslides for a slope-unit to obtain a landslide susceptibility value for that slope-unit. The results of a case study showed that, from a quantitative point of view, integrating spatial probability and size in slope-unit-based landslide susceptibility assessment can bring remarkable increases of AUC (Area under the ROC curve) values. For slope-unit-based scenarios using the logistic regression method and the neural network method, the average increase of AUC brought by incorporating landslide size is up to 0.0627 and 0.0606, respectively. Slope-unit-based landslide susceptibility models incorporating landslide size had utilized the spatial extent information of historical landslides, which was dropped in models not incorporating landslide size, and therefore can make potential improvements. Nevertheless, additional case studies are still needed to further evaluate the applicability of the proposed approach.

## 1. Introduction

Landslide is a phenomenon in which a mass of rock, debris, or earth moves down a slope [1,2]. Landslides can cause huge losses of life and economy; about 14% of total casualties from natural disasters are caused by landslides [3]. Therefore, landslide risk assessment and mitigation are of great significance for human beings. Landslide susceptibility assessment is an essential step of landslide risk assessment [4], thus had been one of the major focuses of landslide studies [5,6,7]. China is particularly badly affected by fatal landslides [8]. Major regions severely affected by landslides in China include the southwest mountainous area [9], southeast coastal area [10], and the loess plateau [11]. Landslide studies in China had focused on various aspects [12]. In particular, landslides induced by the 2008 Wenchuan earthquake had been comprehensively investigated in various studies [13,14,15,16,17], in which landslide susceptibility mapping is also a major focus [18].

Landslide susceptibility, regardless of the type of landslide, is a quantitative or qualitative measurement of the spatial distribution and sizes (e.g., volumes or areas) of landslides which exist or potentially may occur in an area [4]. Therefore, spatial probability and size are two essential components of landslide susceptibility.

Slope-unit (SU) and regular grid are two major types of units used in landslide susceptibility mapping. Slope-unit is a hydrological region bounded by drainage (stream or valley) lines and divide (ridge) lines [19]. Slope-units somehow constrain the extents of gravitational mass flows on slopes, thus are more physically relevant than grid cells. Slope-units had been adopted in many landslide susceptibility mapping applications [19,20,21,22,23,24,25,26,27,28,29,30,31,32,33,34,35] and had been proved to perform better than grid cells in some studies [36,37]. The (total) size of landslides occurring in a particular slope-unit can be accepted as an attribute of this slope-unit as the spatial distribution of a landslide is commonly constrained by the slope-unit in which this landslide is located. By using slope-unit as landslide susceptibility mapping unit, not only the occurrence probability of landslides for a mapping unit (i.e., landslide spatial probability) can be described, but also the size of landslides within this mapping unit can be quantified and predicted.

However, in existing slope-unit-based landslide susceptibility assessments, landslide size is still not taken into account. This is because existing slope-unit-based approaches do not use “the size of landslides that occur in a slope-unit” as a dependent variable to develop and train the landslide susceptibility model. Most slope-unit-based approaches only use “whether a slope-unit has landslides or not” as the dependent variable to develop a landslide susceptibility model [19,20,21,22,23,24,38,39]. That means slope-units are categorized into two classes: slope-units that have landslides and slope-units that do not have landslides. The most commonly used strategy of classification takes the ratio of the (total) area of landslides that occur in a slope-unit to the area of that slope-unit (*AL_SU_/A_SU_*) as a criterion. Those slope-units with *AL_SU_/A_SU_* lower than a predefined threshold are regarded as slope-units without landslide, while those slope-units with *AL_SU_/A_SU_* larger than the predefined threshold are regarded as slope-units with landslide [19,20,39,40,41,42,43,44,45]. In other words, landslide size information is not utilized and predicted in existing slope-unit-based landslide susceptibility models.

The objective of this paper is to present a novel slope-unit-based approach for landslide susceptibility assessment that explicitly integrates landslide spatial probability and size. This novel approach not only predicts the occurrence probability of landslides in a slope-unit but also predicts the size of landslides within that slope-unit. By combing (multiplying) the predicted occurrence probability and size of landslides for a slope-unit, this approach assesses susceptibility integrating spatial probability and size for that slope-unit. First, the procedures of the proposed approach are introduced. Then, the performance of the proposed approach is evaluated and discussed based on a case study.

## 2. Procedures

**Figure 1:**
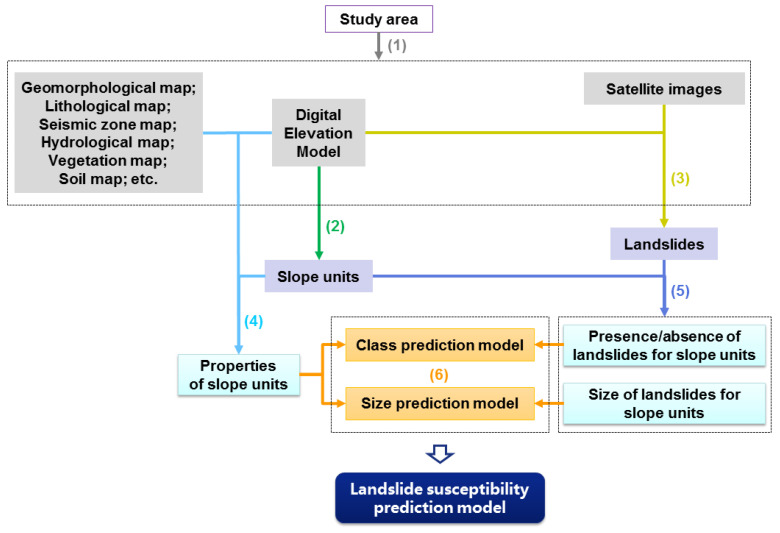
The procedures of the approach for landslide susceptibility assessment integrating spatial probability and size proposed in this paper.

The procedures of the proposed approach for landslide susceptibility assessment proposed in this paper can be divided into six steps, namely data collection and preparation, delineation of slope-units, delineation of landslides, preparation of explanatory variables, preparation of response variables, and generation of prediction models. All the six steps of the proposed approach are shown in Figure 1 and are detailed as follows.

(1) Data collection and preparation. The first step is to collect and prepare data for the study area. Generally speaking, digital elevation model (DEM) and satellite images are obligatory for slope-unit delineation and landslide delineation, respectively. Nevertheless, it is worth noting that DEM also can facilitate and support landslide mapping, and sometimes it is also practical to map landslides using only high-resolution DEM derived from the airborne LiDAR scanning [46] or other methods. Other data characterizing the geo-environmental attributes of the study area, such as a geomorphological map, lithological map, seismic zone map, hydrological map, soil map, vegetation map, are not obligatory but are important for getting a more comprehensive and explanative landslide susceptibility model.
(2) Delineation of slope-units. Slope-units can be broadly regarded as “half basins”, and can be delineated based on DEM using hydrological analysis. A frequently used method to delineate slope-units is intersecting the basin polygons derived from the original DEM and the inverse DEM [23,47,48,49,50]. Slope-units can also be delineated based on curvature [51,52,53]. There is also software that can automatically delineate slope-units [45,54].
(3) Delineation of landslides. Landslides can be delineated either by visual (manual) interpretation or semi-automatic recognition. Although representing landslides using single polygons is acceptable for most applications of susceptibility analysis, explicitly mapping the different engineering geomorphological zones of landslides such as the failure, transition, and deposition areas can promote the analysis of landslide processes and mechanisms [55,56], especially for complex large landslides [57].
(4) Preparation of explanatory variables. As slope-units are used as landslide susceptibility mapping units, explanatory variables are those characterizing the properties of slope-units. Explanatory variables can be either “statistical indices” or “direct indices”. “Statistical indices” are statistics of the values of a certain geo-environmental factor within a certain slope-unit. For factors with numerical values, for example topographic elevation and slope angle, commonly used statistics are mean and standard deviation [20,21,39,43,48,50,58]. Range [44,58] and percentiles [22] were also adopted by several studies. For factors with categorical values, for example, lithology and land cover, both the proportions of each category [20,22,43,45] or the category with the largest proportion [21,58] had been used as explanatory variables. “Direct indices” are parameters characterizing the overall properties of a certain slope-unit, for example, the size, shape, orientation, morphology, bedding attitude, and hydrological properties [20,21,22,43,58].
(5) Preparation of response variables. As both spatial probability and landslide size will be predicted in the proposed approach, response variables are the presence/absence of landslides and the size of landslides in a slope-unit. Those slope-units having one or more than one landslide will be considered unstable (with landslides), while those slope-units having no landslides will be considered stable (without landslides). This criterion is slightly different from the commonly used strategy which defines the presence/absence of landslides based on a non-zero ratio of the area of landslides within a slope-unit to the area of that slope-unit [19,20,39,40,41,42,43,44,45]. The size of landslides in a slope-unit will be the total size of all the landslides within that slope-unit. It must be emphasized that a slope-unit can have more than one landslide. For example, an elongated steep cliff can have many separate failures at different parts on that cliff. In addition, the size of the largest landslide in a slope-unit can be also used as a response variable. This alternative is not trivial when considering that one large landslide is more hazardous than many small ones. Nevertheless, this research adopts the total size of all the landslides within a slope-unit, as this research discusses landslide susceptibility but not the “susceptibility of the largest landslide”.
(6) Generation of prediction models. The proposed landslide susceptibility prediction model consists of two models: a “class prediction model” and a “size prediction model”. For a certain slope-unit, the “class prediction model” gives the probability that this slope-unit has landslides (*PL_SU_*), while the “size prediction model” gives the possible area of landslides within this slope-unit (*AL_SU_*). The final landslide susceptibility index for a slope-unit (*LSI_SU_*) will be the product of the predicted probability of landslide presence and the predicted size of landslides for that slope-unit:
  *LSI_SU_* = *PL_SU_* × *AL_SU_*, (1)

Correspondingly, the final landslide susceptibility index for a grid cell (*LSI_Cell_*) in a certain slope-unit will be the final landslide susceptibility index for that slope-unit divided by the number of grid cells in that slope-unit (*NC_SU_*):

*LSI_Cell_* = *LSI_SU_*/*NC_SU_* = *PL_SU_* × *AL_SU_*/*NC_SU_* = *PL_Cell_* × *AL_Cell_*, (2)

in which

*AL_Cell_* = *AL_SU_*/*NC_SU_*, (3)

is the “average” possible area of landslides per grid cell in this slope-unit, and

*PL_Cell_* = *PL_SU_*, (4)

is the probability that a grid cell in this slope-unit has landslides. It is worth noting that the predicted area of landslides for a slope-unit (*AL_SU_*) cannot be larger than the area of that slope-unit (*A_SU_*). That means, the range of *AL_SU_* will be (0, *A_SU_*], and the range of *AL_Cell_* will be (0, *A_Cell_*], in which *A_Cell_* is the area of a grid cell. As the range of *PL_SU_* is (0, 1), the range of *LSI_SU_* will be (0, *A_SU_*), and the range of *LSI_Cell_* will be (0, *A_Cell_*). The prediction models can be implemented using many methods, such as logistic regression and neural networks.

It must be emphasized that step 1 to step 5 are basic steps for all slope-unit-based approaches for landslide susceptibility assessment, in which explanatory and response variables for landslide susceptibility prediction models are prepared. The difference in the novel approach proposed in this paper compared with previous approaches lies in the last step in which prediction models are generated. Previous slope-unit-based approaches only predict the occurrence probability of landslides by generating a “class prediction model”, while this novel approach also predicts the size of landslides by generating a “size prediction model” and further multiplies the predicted occurrence probability and size of landslides for a slope-unit.

## 3. Case Study

### 3.1. Study Area and Data

**Figure 2:**
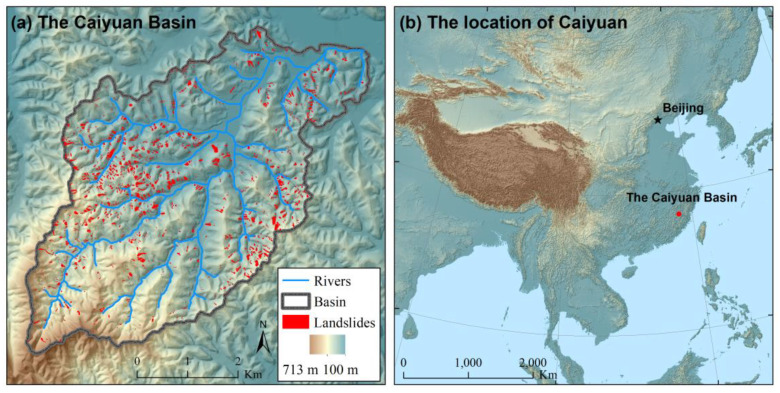
The Caiyuan Basin (**a**) as well as its location in East Asia (**b**). The delineated landslides in the Caiyuan Basin are also shown in (**a**).

**Figure 3:**
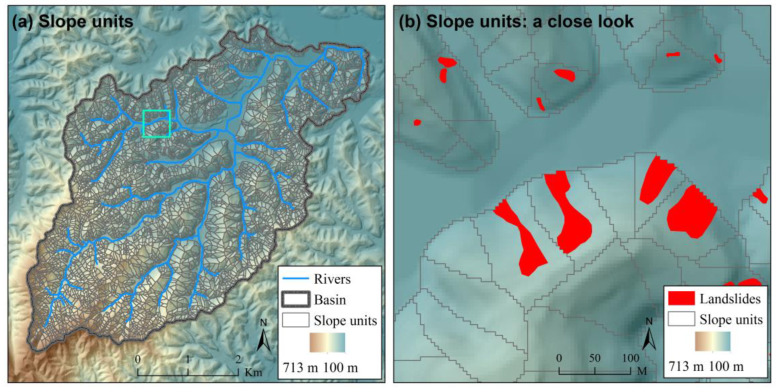
The delineated slope-units in the Caiyuan Basin (**a**) with a close look (**b**). The extent of the close look in (**b**) is shown in (**a**) with a cyan square.

The Caiyuan Basin in Fujian Province, China (Figure 2) has been chosen to evaluate the proposed approach for landslide susceptibility assessment that explicitly integrates landslide spatial probability and size. The Caiyuan Basin is highly prone to landslides due to rugged topography and frequent storm rainfalls. Large numbers of landslides occurred in the Caiyuan Basin during a storm rainfall event in June 2010. First, slope-units were delineated based on a 5 m × 5 m spatial resolution DEM with the automatic software r.slopeunits [45] and the assistance of manual editing of the automatic generated results (Figure 3). Then, with the support of topographic hillshade maps and slope-unit data, landslides were manually delineated on 2.5 m × 2.5 m spatial resolution SPOT images taken shortly after the storm rainfall event (Figure 2a). Landslides were represented by single polygons embracing both their source zones and transition-deposition zones. Field surveys [59] confirm that most of the landslides in the study area are shallow earth slides that do not cross over slope-unit boundaries.

### 3.2. Scenarios

There are three fundamental types of methods for landslide susceptibility assessment [4], namely the qualitative “knowledge-driven methods” [60,61,62], quantitative “data-driven methods” [24,63,64], and quantitative “physically based methods” [65,66,67]. Generally, more complex methods that require a larger amount of and more detailed data are applied to landslide susceptibility assessment of larger scales [68]. Data-driven methods (e.g., statistical methods) evaluate landslide susceptibility by referring to the geoenvironmental characteristics of those locations where landslides had occurred [7], and have become standard in regional-scale landslide susceptibility mapping [4]. Most published works on landslide susceptibility had implicitly used data-driven methods [63,69,70,71,72,73,74,75,76,77,78,79,80,81,82,83].

Three data-driven methods, namely the logistic regression (LR) method, neural network (NN) method, and a modified frequency ratio (FR) method [7], were used for predicting the spatial probability of landslides. The neural network method was used for predicting the size (area) of landslides. Totally seven scenarios of landslide susceptibility assessment were implemented in the case study to evaluate the proposed approach (Table 1). Three scenarios, namely “Grid (FR)”, “Grid (LR)” and “Grid (NN)”, used the regular grid as a mapping unit. Four scenarios, namely “SU (LR)”, “SU (NN)”, “SU (LRNN)” and “SU (NNNN)”, used slope-unit as mapping unit. Two of the scenarios based on slope-units, namely “SU (LRNN)” and “SU (NNNN)”, had explicitly incorporated landslide size, while the other five scenarios predicted only landslide spatial probability. For grid-based scenarios, seven landslide-related factors were adopted to develop a landslide susceptibility model, namely elevation, slope angle, slope aspect, standard curvature, plan curvature, profile curvature, and topographic wetness index. For slope-unit based scenarios, totally forty-one explanatory variables were adopted, including thirty-eight “statistical indices” and three “direct indices”. Details of the explanatory variables are illustrated in Table 1. All these landslide-related factor data were derived from the 5 m × 5 m spatial resolution DEM. Only factors derived from DEM were used because other geo-environmental data, such as a geological map and vegetation map, with adequately large scale and spatial resolution are not available for the Caiyuan Basin. Because random processes are involved in the logistic regression method and neural network method, each of the six scenarios using the two methods was implemented 100 times.

## 4. Results and Discussion

### 4.1. Results

The performances of different landslide susceptibility models were evaluated using ROC (receiver operating characteristic) curve. A ROC curve [84] is a graphical plot used to select the best performance classifier model or to detect the optimal discrimination threshold of a classifier model. A ROC curve is created by plotting the true positive rate (TPR) against the false positive rate (FPR) at various discrimination thresholds. The point with a 0 FPR and a 1 TPR at the top left corner of the ROC space is called a perfect classification, which means that predictions are 100% right. A model with a larger AUC (Area under the ROC Curve) is closer to the perfect classification point and thus performs better.

**Figure 4:**
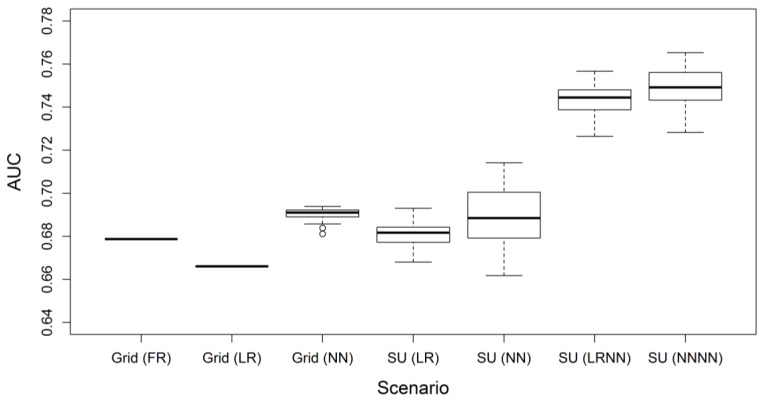
The boxplot of the AUCs for different scenarios of landslide susceptibility assessment in the case study. Scenario “Grid (FR)” only has one simulation because it does not involve random processes. Each of all other scenarios has 100 stochastic simulations. The boxplot for scenario “Grid (LR)” looks like a single line, since the variation of its AUCs is negligible. It is obvious that scenario “SU (LRNN)” and “SU (NNNN)” incorporating landslide size have AUCs remarkably higher than that of other scenarios. Detailed information about the scenarios is referred to in Table 1.

The boxplot of the AUCs for different scenarios of landslide susceptibility assessment is shown in Figure 4. The AUC for scenario “Grid (FR)” is 0.6787. The average AUCs in the 100 Monte Carlo simulations for scenario “Grid (LR)”, “Grid (NN)”, “SU (LR)”, “SU (NN)”, “SU (LRNN)” and “SU (NNNN)” are 0.6660, 0.6904, 0.6808, 0.6889, 0.7435 and 0.7495, respectively. It is obvious that scenarios explicitly incorporating landslide size, i.e., scenario “SU (LRNN)” and “SU (NNNN)”, have the highest AUCs and therefore the best performance compared with other scenarios. While scenario “Grid (FR)”, “Grid (LR)”, “Grid (NN)”, “SU (LR)” and “SU (NN)” have similar AUCs, scenario “SU (LRNN)” and “SU (NNNN)” have much larger AUCs. For slope-unit-based scenarios using the logistic regression method, the lowest increase of AUC brought by incorporating landslide size is 0.0444, and the average is up to 0.0627. For slope-unit-based scenarios using the neural network method, the lowest increase of AUC brought by incorporating landslide size is 0.0389, and the average is up to 0.0606.

**Figure 5:**
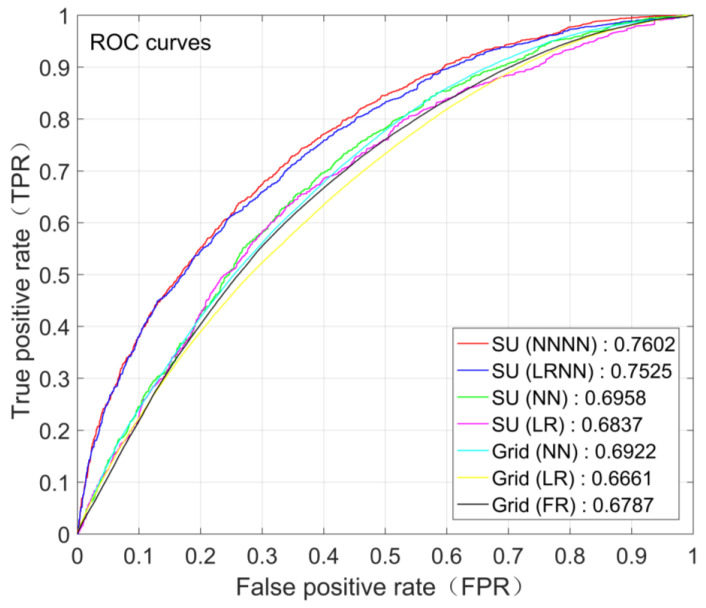
The ROC curves for different scenarios of landslide susceptibility assessment in the case study. The ROC curves for scenario “Grid (LR)”, “Grid (NN)”, “SU (LR)”, “SU (NN)”, “SU (LRNN)” and “SU (NNNN)” show the results of one of the 100 Monte Carlo simulations in the case study. Detailed information about the scenarios are referred to in Table 1.

**Figure 6:**
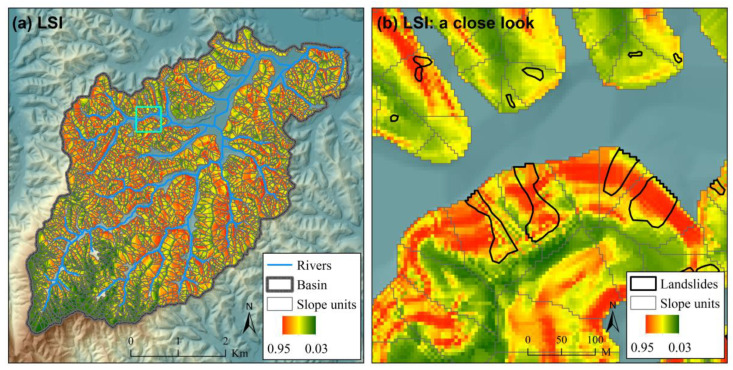
The landslide susceptibility index (*LSI*) for scenario “Grid (NN)” in the case study (**a**) with a close look (**b**). The extent of the close look in (**b**) is shown in (**a**) with a cyan square. This figure shows the result of one of the 100 Monte Carlo simulations in the case study, which is the same as that in Figure 5. Detailed information about the scenarios are referred to in Table 1.

**Figure 7:**
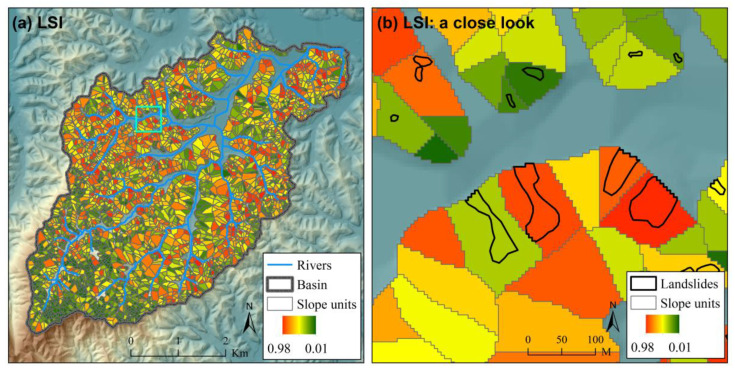
The landslide susceptibility index (*LSI*) for scenario “SU (NN)” in the case study (**a**) with a close look (**b**). The extent of the close look in (**b**) is shown in (**a**) with a cyan square. This figure shows the result of one of the 100 Monte Carlo simulations in the case study, which is the same as that in Figure 5. Detailed information about the scenarios are referred to in Table 1.

**Figure 8:**
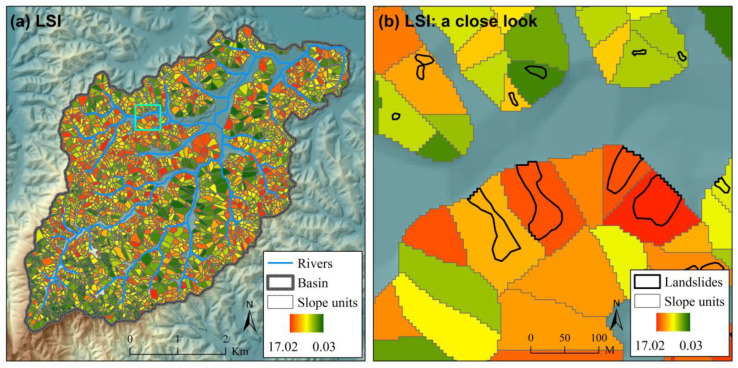
The landslide susceptibility index (*LSI*) for scenario “SU (NNNN)” in the case study (**a**) with a close look (**b**). The extent of the close look in (**b**) is shown in (**a**) with a cyan square. This figure shows the result of one of the 100 Monte Carlo simulations in the case study, which is the same as that in Figure 5. Detailed information about the scenarios are referred to in Table 1.

**Figure 9:**
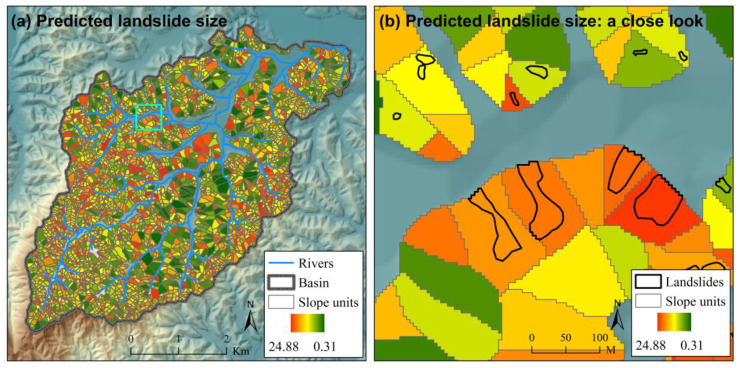
The predicted landslide size (area) for each grid cell in the case study (**a**) with a close look (**b**). The extent of the close look in (**b**) is shown in (**a**) with a cyan square.

The ROC curves for different scenarios of landslide susceptibility assessment in the case study are shown in Figure 5. The ROC curves for scenarios expect “Grid (FR)” to show the results of one of the 100 Monte Carlo simulations. For illustration, the spatial distributions of landslide susceptibility index (*LSI*) for scenario “Grid (NN)”, “SU (NN)” and “SU (NNNN)” are also shown in Figure 6, Figure 7 and Figure 8 (the results of the same Monte Carlo simulation as that in Figure 5 are shown). The predicted landslide size (area) for each grid cell in the case study is shown in Figure 9. The maximum predicted landslides area for a grid is 24.88 m^2^, which does not exceed the area of a grid (5 m × 5 m). For each grid cell, the landslide susceptibility index shown in Figure 8 is the product of the landslide susceptibility index (predicted spatial probability) shown in Figure 7 and the predicted landslide size shown in Figure 9.

### 4.2. Explanation of Results

The case study shows that for landslide susceptibility models using the same method, the change of the mapping unit for the regular grid to slope-unit does not bring significant changes to the model performances. On the contrary, the incorporation of landslide size does bring remarkable increases of AUC to landslide susceptibility models. This finding implies that incorporating landslide size can significantly improve slope-unit-based landslide susceptibility assessment. The reason for this phenomenon is that the slope-unit-based landslide susceptibility model incorporating landslide size involves all the information provided by landslide data. On the contrary, the slope-unit-based landslide susceptibility model not incorporating landslide size drops the spatial extent information of landslide provided by landslide data. Specifically, both the presence/absence of landslides and the size of landslides are attributes of slope-units. However, in the landslide susceptibility model not incorporating landslide size, all slope-units with landslides will be categorized as the same class, although they may have different landslide sizes.

Landslide susceptibility is also considered to be the probability that an area has a landslide [5]. This definition of landslide susceptibility does not involve landslide size and is different from the definition adopted in this paper [4]. Considering that different mapping units can be used in landslide susceptibility assessment, a more general definition is believed to be the one regarding landslide susceptibility as a measurement of both the spatial probability and sizes of landslides. For regular grid mapping units, involving landslide size in susceptibility or not are equivalent. This is because a grid is predicted to have landslides means that the whole grid will be covered by landslides. The predicted landslide sizes for all grids will be the same since all regular grids have the same area. In other words, for regular grid mapping units, only considering spatial probability in landslide susceptibility will be adequate, while considering both spatial probability and size in landslide susceptibility will not help to increase the success of its assessment. However, for the slope-unit mapping unit, not involving size in landslide susceptibility will be questionable. The probability that a slope-unit has a landslide solely cannot describe the spatial extent of potential landslides in that slope-unit, and therefore cannot represent the “real spatial distribution” of potential landslides.

### 4.3. Noncollinearity between Spatial Probability and Size

A prerequisite for this procedure is that the probability of landslide presence (*PL_SU_*) and the size of potential landslides (*AL_SU_*) for a slope-unit are not collinear (not correlated). Intuitive speculation is that the two variables are positively correlated, i.e., slope-units having larger *PL_SU_* will have larger *AL_SU_*. Nevertheless, an opposite question would be if it is physically possible for *PL*_SU_ and *AL_SU_* to be “negatively correlated”. Although it is hard to find real cases to directly show the “negative correlation” between *PL_SU_* and *AL_SU_*, theoretical reasoning might help to understand the possibility. Considering a situation in which a rigid block is placed on an inclined plane. If we regard the rigid block as a slope-unit, the possible area of landslides within this slope-unit (*AL_SU_*) would be equal to the area of the block since this block is rigid. In the real world, this situation means the “average” possible area of landslides per grid cell in this slope-unit (*AL_Cell_*) would be equal to the area of a grid cell (*A_Cell_*). In other words, the size of potential landslides within this slope-unit has a maximum value. Despite this, the probability that this slope-unit has landslides (*PL_SU_*), i.e., the probability that the rigid block slides down the inclined plane, can be small if the angle of the inclined plane is small. It is worth noting that, the probability that the rigid block slides along the inclined plane cannot be zero even if the angle of the inclined plane is zero. This is because theoretically shaking forces from an earthquake can make a block resting on a horizontal plane move. This theoretical reasoning shows the possibility of large *AL_SU_* coupled with small *PL_SU_*. Similarly, small *AL_SU_* coupled with large *PL_SU_* is also physically possible. Considering slope-unit, most of which is stable, while a small part of which is highly prone to failure. Then, this slope-unit will be expected to have large *PL_SU_* and small *AL*_SU_. As both positively correlated and negatively correlated are possible, *PL_SU_* and *AL_SU_* are not expected to be correlated.

**Figure 10:**
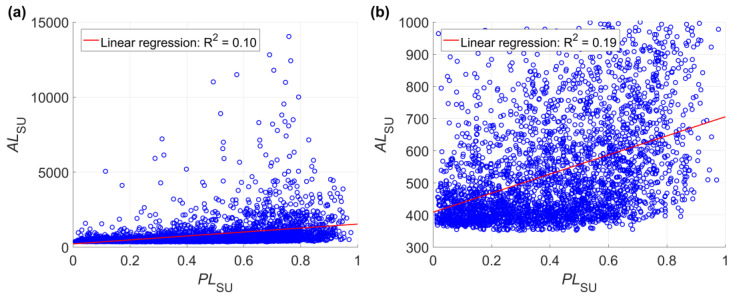
Linear regressions between *PL_SU_* and *AL*_SU_ based on all values of *AL_SU_* (**a**) and values of *AL_SU_* below 1000 (**b**). This figure shows the result of one of the 100 Monte Carlo simulations in the case study, which is the same as that in Figure 5. Detailed information about the scenarios are referred to in Table 1. This figure shows the values of *PL_SU_* predicted using the neural network method, and values of *PL_SU_* predicted using the logistic regression method show similar behaviors.

**Figure 11:**
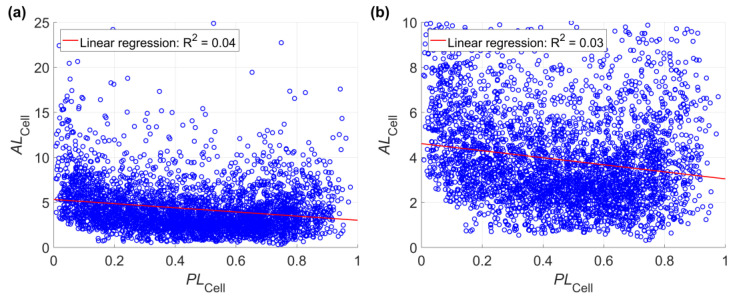
Linear regressions between *PL_Cell_* (*PL_SU_*) and *AL_Cell_* based on all values of *AL_Cell_* (**a**) and values of *AL_Cell_* below 10 (**b**). This figure shows the result of one of the 100 Monte Carlo simulations in the case study, which is the same as that in Figure 5. Detailed information about the scenarios are referred to in Table 1. This figure shows the values of *PL_Cell_* predicted using the neural network method, and values of *PL_Cell_* predicted using the logistic regression method show similar behaviors.

The noncollinearity between *PL_SU_* and *AL_SU_* was also validated by the numerical predictions in the case study. According to Figure 10, fitting linear regressions between *PL_SU_* and *AL_SU_* will get very low coefficients of determination. Similarly, collinearity between *PL_Cell_* (*PL_SU_*) and *AL_Cell_* is also not observed (Figure 11). The noncollinearity between *PL_SU_* and *AL_SU_* leads to the necessity of integrating landslide probability and landslide size. Because of the noncollinearity between *PL_SU_* and *AL_SU_*, only considering *PL_SU_* or only considering *AL_SU_* in landslide susceptibility will be potentially biased.

### 4.4. Applicability of Incorporating Size

The application of the proposed approach for landslide susceptibility assessment considering landslide size, however, is not straightforward. First, data with high resolution and high accuracy are required. For example, the delineation of slope-units should be on a proper scale so that the delineated slope-units can be broadly regarded as “meta-units” in which landslides occur. Slope-units delineated based on low-resolution DEM is just “half basins”. DEM with adequately high resolution is required for generating slope-units with an adequately fine scale. Second, the method is not applicable, at least directly, for all landslide types. As it was already mentioned it cannot be applied for large deep-seated landslides whose source zones can cross the watersheds. Besides, it requires some modification for very long runout types such as rock avalanches and debris flows whose transition and deposition zones can extend outside the single slope-unit where they are sourced. Moreover, additional results from other case studies are needed to further evaluate the applicability of the proposed approach.

## 5. Conclusions

This paper presented a novel slope-unit-based approach for landslide susceptibility assessment that explicitly integrates landslide spatial probability and size. The proposed approach uses slope-unit as a landslide susceptibility mapping unit. Previous slope-unit-based approaches only predict the occurrence probability of landslides by generating a “class prediction model”, while this novel approach also predicts the size of landslides by generating a “size prediction model” and further multiplies the predicted occurrence probability and size of landslides to get a landslide susceptibility value for a slope-unit. Therefore, the landslide susceptibility value for a slope-unit given by this novel approach is an integration of the predicted occurrence probability (spatial probability) of landslides and predicted size (area) of potential landslides for that slope-unit.

A case study was implemented to evaluate the proposed approach. The study area was the Caiyuan basin in Fujian Province, China, which distributes large numbers of rainfall-induced landslides. The logistic regression method and the neural network method were used to predict the occurrence probability of landslides, and the neural network method was used to predict the size of landslides. The results showed that integrating spatial probability and size had significantly improved slope-unit-based landslide susceptibility assessment. A series of Monte Carlo simulations showed that the average increase of AUC brought by incorporating landslide size could be up to 0.06. Specifically, for slope-unit-based scenarios using the logistic regression method and the neural network method, the average increase of AUC brought by incorporating landslide size is up to 0.0627 and 0.0606, respectively.

Landslide sizes quantitatively describe the spatial extents of historical landslides. Therefore, considering landslide size together with landslide spatial probability in slope-unit-based methods can bring potential improvements to landslide susceptibility assessment. Nevertheless, the applicability of the proposed approach needs further evaluations based on other case studies. In addition, complicated by varieties of data availability and landslide characteristics, the application of the proposed approach for landslide susceptibility assessment considering landslide size is not straightforward.

## Figures and Tables

**Table 1:** Scenarios of landslide susceptibility assessment in the case study.

Scenario	Unit ^a^	Method ^b^		Explanatory Variable ^c^
		Probability	Size	
Grid (FR)	Grid	FR	N.A.	H, AN, AS, SC, PLC, PRC, TWI
Grid (LR)	Grid	LR	N.A.	
Grid (NN)	Grid	NN	N.A.	
SU (LR)	SU	LR	N.A.	H_Min_, H_Max_, H_Range_, H_Mean_, H_Std_, H_Sum_
SU (NN)	SU	NN	N.A.	AN_Min_, AN_Max_, AN_Range_, AN_Mean_, AN_Std_, AN_Sum_
SU (LRNN)	SU	LR	NN	AS_Mean_, AS_Std_
SU (NNNN)	SU	NN	NN	SC_Min_, SC_Max_, SC_Range_, SC_Mean_, SC_Std_, SC_Sum_ PLC_Min_, PLC_Max_, PLC_Range_, PLC_Mean_, PLC_Std_, PLC_Sum_ PRC_Min_, PRC_Max_, PRC_Range_, PRC_Mean_, PRC_Std_, PRC_Sum_ TWI_Min_, TWI_Max_, TWI_Range_, TWI_Mean_, TWI_Std_, TWI_Sum_ P, A, SI

^a^ Unit: “Grid” and “SU” mean regular gird and slope-unit are used as landslide susceptibility mapping units, respectively. ^b^ Method: The column “Probability” indicates the method used for predicting the spatial probability of landslides. The column “Size” indicates the method used for predicting the size (area) of landslides. “FR”, “LR” and “NN” mean frequency ratio, logistic regression, and neural network method, respectively. “N.A.” means not applicable. ^c^ Explanatory variable: “H”, “AN”, “AS”, “SC”, “PLC”, “PRC”, “TWI” mean elevation, slope angle, slope aspect, standard curvature, plan curvature, profile curvature, and topographic wetness index, respectively. For slope-unit-based scenarios, 38 statistical indices were adopted. Subscript “Min”, “Max”, “Range”, “Mean”, “Std” and “Sum” mean the minimum, maximum, range, mean, standard deviation, and summation of factor values within a slope-unit. Slope aspect only have mean and standard deviation statistics because it is a circular quantity. “P”, “A” and “SI” are 3 direct indices for slope-unit-based scenarios, and mean perimeter, area, and shape index of a slope-unit, respectively.
